# Genetic and Environmental Influences on Longitudinal Trajectories of Functional Biological Age: Comparisons Across Gender

**DOI:** 10.1007/s10519-017-9851-5

**Published:** 2017-05-27

**Authors:** Deborah Finkel, Ola Sternäng, Åke Wahlin

**Affiliations:** 1 Department of Psychology, Indiana University Southeast, New Albany, USA; 2 Institute of Gerontology, School of Health and Welfare, Jönköping University, Box 1026, 551 11 Jönköping, Sweden; 3 Department of Psychology, Stockholm University, Stockholm, Sweden

**Keywords:** Functional bioage, Chronological age, Twins

## Abstract

We used an alternate age variable, functional biological age (fBioAge), which was based on performance on functional body measures. The aim was to examine development of fBioAge across the adult life span, and to also examine potential gender differences and genetic and environmental influences on change with age. We used longitudinal data (*n* = 740; chronological age (ChronAge) range 45–85 at baseline) from the Swedish Adoption/Twin Study of Aging. The rate of increase in fBioAge was twice as fast after ChronAge 75 than before. fBioAge was higher in women than in men. fBioAge was fairly equally influenced by genetic and environmental factors. Whereas the rate of ChronAge cannot vary across time, gender, or individual, our analyses demonstrate that fBioAge does capture these within and between individual differences in aging, providing advantages for fBioAge in the study of aging effects.

## Introduction

In most aging studies, chronological age (ChronAge) has been used as the aging indicator. However, ChronAge only measures how much time that has passed since birth. The definition of ChronAge suggests that the aging processes take place along a continuum with exact distances, across the whole adult lifespan. However, the fact that one chronological year has passed, does not necessarily mean that an individual also has aged the equivalent of 1 year. Probably, the speed of the aging processes both vary and differ during the adult lifespan, such that an individual may age more during certain periods and less during other periods. Also, the effects of aging are to a large extent individual. For example, a group of randomly selected older persons with a ChronAge of 75 can be rather heterogeneous on a particular aspect of aging (Gunn et al. 2009). It would, therefore, be more reasonable to use an age measure that better captures aging, per se. No single biomarker (e.g., grip strength or lung function) has been found to alone reflect the aging processes of the body in a representative way. Often researchers use selected samples of biomarkers to better capture these complex processes across time. No standard combination is used, but if different operational definitions of a construct are used, then also a more nuanced picture of the phenomenon (in this case age/aging and its connection to other variables) will be provided.

In the present longitudinal study, we used an age variable named functional biological age (fBioAge), which is based on performance on functional body measures, i.e. grip strength, lung functioning, gait, visual acuity, and auditory acuity (Anstey et al. 1996). Functional biomarkers measure behavior and perception and are, therefore, relevant for the study of psychology and aging (Anstey 2008), and in this study, our ambition was to use an age indicator relevant for research in cognitive aging. Previous analyses of fBioAge in this sample (Sternäng et al. under review) indicate that it meets many of the criteria for a good indicator of aging, as outlined by McClearn (1997): it changes at a rate that reflects the rate aging, it reflects physiological age, the relationship with illness and ADL indicates that fBioAge is crucial to the maintenance of health, fBioAge successfully differentiates shorter and longer term survival, and fBioAge demonstrates significant change over a short period of time.

The concept of fBioAge has been used for some time now, and prominently by Anstey (2008). An fBioAge variable has also recently been developed and examined by our own group in a cross-sectional setting (Wahlin et al. under review). The present study takes the topic one step further, by using a longitudinal approach, and by simultaneous examination of both environmental and genetic influences on fBioAge variability.

For this research, we used longitudinal data from the Swedish Adoption/Twin Study of Aging (SATSA; Finkel and Pedersen 2004). The participants in SATSA are identical and fraternal twins, reared apart or reared together, which provides the possibility to examine influences of genetic and environmental factors on individual differences.

The goals of the present study were to examine longitudinal changes in fBioAge across the later parts of the adult life span (ChronAge 50+), to examine potential gender differences in these trajectories, and to examine genetic and environmental influences on individual differences in aging trajectories. The corresponding hypotheses were: (i) The rate of increase in fBioAge will (contrary to ChronAge) vary significantly across the examined ChronAge span. (ii) Gender differences will be found in both levels and rates of change in the fBioAge trajectories. Research has shown that there are gender differences in levels of biological age, health and longevity (Brayne et al. 2001; Nakamura and Miyao 2008; United Nations Statistics Division 2013), and in rates of change in biological age (Nakamura and Miyao 2008). (iii) The influence of environmental factors on fBioAge will increase in old age with higher ChronAge (our study included an age range up to 93 years of age). Studies have shown an increasing influence of environmental factors in old age in, for example, self-rated health (Gavrilova and Gavrilov 2009), motor functioning (Finkel et al. 2015), and cognitive measures (Finkel and Reynolds 2010).

## Methods

### Participants

Accrual procedures for SATSA have been described previously. In brief, the sample is a subset of twins from the population-based Swedish Twin Registry (Finkel and Pedersen 2004). In-person testing (IPT) took place in a location convenient to the participants, such as district nurses’ offices, health-care schools, and long-term care clinics. Testing was completed during a single 4-hour visit. All variables included in the current analyses were collected beginning at the second wave (IPT2). Most waves of IPT occurred at 3-year intervals, with one exception: in-person testing did not occur during wave 4. Therefore, the total time span from IPT2 to IPT8 was 19 years.

In total, data from 740 individuals were included in the current analyses: 304 men and 436 women. Of those participants, 61% provided data at three or more time points and 16% participated at all six waves. Mean number of waves of participation was 3.38 (SD = 1.7) for men and 3.33 (SD = 1.7) for women. Table 1 presents descriptive information at each wave. Age range at each wave is presented; age was fairly normally distributed at each wave. Women averaged 1.5–2 years older than men at each IPT, a difference that was significant at p < .05 at IPT2, IPT5, and IPT7. Using an age-based latent growth curve model (described below) instead of a time-based model ensures that age is equated across sex. Because SATSA is a cohort-sequential design, new participants were added at waves IPT3 through IPT5, and some participants were lost due to attrition (Finkel and Pedersen 2004). Four types of twins participate in SATSA: monozygotic twins reared apart (MZA), monozygotic twins reared together (MZT), dizygotic twins reared apart (DZA), and dizygotic twins reared together (DZT). Number of twin pairs participating at each IPT is indicated in Table 1; data from both complete and incomplete pairs were used in the analyses to maximize power.

**Table 1:** Sample characteristics

Wave	Men				Women			
	N	N pairs	Mean ChronAge (SD)	Mean fBioAge (SD)	N	N pairs	Mean ChronAge (SD)	Mean fBioAge (SD)
			(in years)	(in T scores)			(in years)	(in T scores)
IPT2	225	13/26/24/35	64.7 (8.4)	48.7 (5.3)	317	16/27/56/33	66.3 (9.0)*	55.1 (5.5)**
IPT3	186	11/12/21/31	67.3 (8.7)	48.9 (5.6)	268	9/24/42/26	69.4 (9.4)	55.2 (6.3)**
IPT5	199	9/20/24/25	68.7 (9.3)	50.3 (5.9)	285	17/24/39/25	70.5 (9.7)*	56.1 (6.7)**
IPT6	165	8/14/18/15	70.6 (8.8)	51.4 (5.9)	210	10/17/23/16	71.5 (8.7)	56.2 (5.6)**
IPT7	137	6/11/10/18	72.6 (8.1)	51.0 (5.9)	201	8/15/22/17	74.6 (9.0)*	57.1 (7.5)**
IPT8	117	6/7/9/15	74.1 (7.7)	51.7 (7.7)	171	9/11/19/13	75.4 (8.2)	56.3 (6.1)**

*Note*: N pairs indicates number of monozygotic reared apart/monozygotic reared together/dizygotic reared apart/dizygotic reared together twin pairs

*Mean ChronAge for women is significantly greater than mean ChronAge for men at *p* < .05

**Mean fBioAge for women is significantly greater than mean fBioAge for men at p < .0005

### Measures

#### Vision and hearing

Vision and hearing were measured via self-report. Participants were asked to rate their vision on a scale from 1 (excellent) to 5 (nearly blind or blind) and to rate their hearing on a scale from 1 (excellent) to 5 (nearly deaf or deaf).

#### Muscle strength

The participants’ grip strength was measured by a Collin handgrip dynamometer (0–70 kg) by a trained research nurse at the IPT. The participant made six attempts (three with each hand) and the maximum score (in kg) was considered as the participant’s grip strength score.

#### Walking speed time

Time to walk 3 m and return was recorded starting at IPT2. The nurse administering the interview used a stopwatch to measure the amount of time it took (in seconds) for the participant to complete this task.

#### Lung function

Lung function was tested on one of two inter-calibrated portable 10-1 dry bellows Vicatest spirometers (Mijnhardt, Bunnik, The Netherlands) with subjects in seated position and their nasal passages blocked with nose clips. Forced expiratory volume in the first second (FEV_1_) was used in the current analyses. At IPT7, only one trial was collected; during IPT2 through IPT6 two trials were completed, and data from the best trial were used in the present analyses. During the course of the study, it became necessary to change spirometric equipment, due to the increasing difficulty of transporting the Vicatest spirometers and the availability of new spirometric equipment that was lighter and easier for the nurses to use. Thus, at IPT3, pulmonary function for 30% of the subjects was measured using the Vicatest, and the remaining subjects were assessed with a portable ML 330 spirometer (Micor Medical, Kent, United Kingdom). The two spirometers were inter-calibrated to ensure consistent measurement. FEV_1_ values for both spirometers were expressed in BTPS (body temperature and pressure saturated with water vapor).

#### Functional biological age

The five indicators above used for the construction of fBioAge were significantly intercorrelated. We aimed to examine sex differences in fBioAge beyond the trivial fact that men have greater body mass and stronger muscles in general than women. Before calculation of fBioAge, FEV_1_ was, therefore, corrected for body volume through division by the individual’s squared height (m2). The five variables were then z-transformed separately with IPT2 values as the basis and summed to create a composite score. To correct for sex in the grip strength scores, the z transformation of grip strength was done separately for women and men. The mean fBioAge at each IPT is presented separately for men and women in Table 1.

### Statistical method

**Fig. 1:**
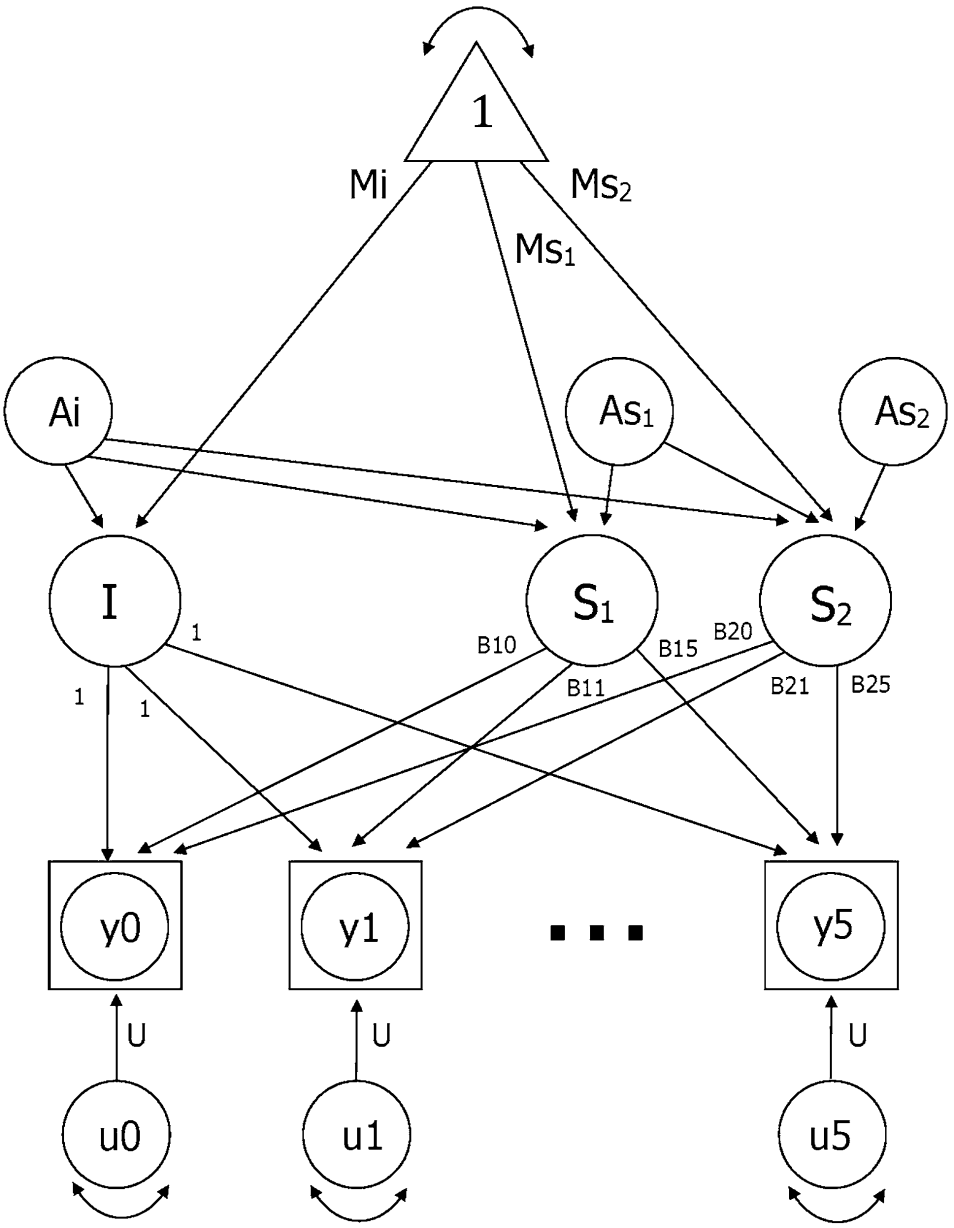
Two-slope latent growth curve model. Note *I* = intercept, *S*_1_ = slope 1, *S*_2_ = slope 2; Observed data are indicated by y0 through y5. Group mean intercept (Mi) and slopes are estimated (Ms1 and Ms2) and residual variances (u0 through u5) are set equal across waves. The paths from the latent slope factors to the observed scores are the age basis coefficients, B1(t) and B2(t). For simplicity, the model includes only the additive genetic effects for the intercept (Ai) and slopes (As1 and As2)

Due to the range in ChronAge at each IPT (up to 40 years) an age-based biometric latent growth curve model (LGCM) was used to examine genetic and environmental contributions to ChronAge changes in fBioAge (Neale and McArdle 2000). Note that age-based and time-based models provided similar results. The LGCM provides estimation of fixed effects, i.e. fixed population parameters as estimated by the average growth model of the entire sample, and random effects, i.e. individual variation in growth model parameters. Comparing a model with one slope to a model with two slopes indicated that the two-slope model provided a better fit to the data (likelihood ratio test = 138.6, df = 4, *p* < .01). Therefore, a two-slope LGCM was used (Finkel et al. 2003): one slope for younger old (ChronAge < 75) and a separate slope for older old (ChronAge > 75). An empirical method was used to determine the best centering age: models centered at different ages (60, 65, 70, 75, 80) were compared. Because the models are not nested, we could not compare fits via a likelihood ratio test. Instead we examined Aikaike’s Information Criterion and the residual variance estimated by the model. In the model with centering age set of ChronAge 75, AIC and residual variance were both minimized, indicating that the age-75 model produced the best fit to the data. Thus, the intercept is evaluated at the inflection point: ChronAge 75. The age-based latent growth curve model is presented in Fig. 1. Observed data are indicated by y0 through y5. Group mean intercept (Mi) and slopes are estimated (Ms_1_ and Ms_2_) and residual variances (u0 through u5) are set equal across waves. The paths from the latent slope factors to the observed scores are the age basis coefficients, B1(t) and B2(t). The age basis serves as a marker for the age of the subject at each time of measurement, adjusted for the centering age. Therefore, age basis coefficients are defined as an individual’s observed ChronAge at each measurement occasion minus the centering age (75 years). Values of B1(t) were set to zero for any ChronAge greater than 75, thereby defining S1 as the rate of change up to ChronAge 75. Similarly, values of B2(t) were set to zero for any ChronAge less than 75, defining S2 as the rate of change after ChronAge 75. When the age basis is set to zero, the individual cognitive score associated with that measurement occasion does not contribute to the estimation of the particular slope (i.e., the slope prior to ChronAge 75 or alternatively after ChronAge 75).

Using twin data, the random effects, or variance, in latent growth curve parameters can be divided into four separate components: additive genetic effects (A), correlated environmental effects shared by anyone living in the same culture (C), shared rearing environmental effects shared only by twins who grew up in the same home (S), and nonshared environmental effects unique to each individual and error associated with age-specific residuals (E). For simplicity, the model in Fig. 1 includes only the additive genetic effects for the intercept (Ai) and slopes (As_1_ and As_2_). Genetic influences on correlations among intercepts and slopes, are captured by the paths from Ai to S_1_ and S_2,_ and from As_1_ to S_2_. In total then, there are six genetic parameters (paths) estimated by the model. Shared rearing environment, correlated environment, and nonshared environment were also included in the model, for a total of 24 biometric parameters.

By fitting structural models to the observed MZA, MZT, DZA, and DZT covariance matrices, we can estimate the proportion of phenotypic variance accounted for by the variance in genetic factors, shared environment factors, correlated environment factors, and nonshared environment factors. Biometric latent growth curve models were fit with the structural equation modeling program Mx version 1.66b (Neale et al. 2003). The raw maximum likelihood estimation procedure was used throughout. We tested nested models using a likelihood ratio test (i.e. subtracting the −2 log likelihoods of the models being compared).

## Results

### Model comparisons

In the first set of models, sex differences in the biometric latent growth curve model were tested, as reported in the top of Table 2. First, the full model with all parameters estimated separately for men and women was fit to the data. In model 2, all model parameters were equated across sex: three growth curve parameters (intercept, slope 1, and slope 2) and 24 biometric parameters (paths for A, C, S, and E). The likelihood ratio test (LRT) comparing the fit of model 2 to model 1 indicated a significant reduction in model fit. In model 3, only the three growth parameters were equated across sex, which also resulted in significant reductions in model fit versus model 1. When only the biometric parameters were equated across sex in model 4, no reductions in model fit occurred. Thus, men and women differ in the shape of the change trajectory, but not in genetic and environmental influences on that trajectory.

**Table 2:** Model-fitting results

Model	−2LL (df)	LRT (df)
Initial model testing (vs. model 1)		
1. Full model	13252.9 (2339)	
2. Equate all across sex	13446.4 (2366)	193.5 (27)**
3. Equate LGCM across sex	13421.6 (2342)	168.7 (3)**
4. Equate biometric across sex	13261.7 (2363)	8.7 (24)
Follow-up testing of LGCM (vs. model 4)		
5. Equate I across sex	13373.5 (2364)	111.8 (1)**
6. Equate S_1_ across sex	13262.8 (2364)	1.2 (1)
7. Equate S_2_ across sex	13264.1 (2364)	2.4 (1)
Follow-up testing of biometric (vs. model 4)		
8. Drop A both sexes	13270.4 (2369)	8.8 (6)
9. Drop CS both sexes	13267.6 (2375)	14.7 (12)
10. Drop ACS both sexes	13294.3 (2381)	41.4 (18)**

*LRT* Likelihood ratio test

**Difference in model fit is significant at *p* < .01

### Latent growth curve

**Fig. 2:**
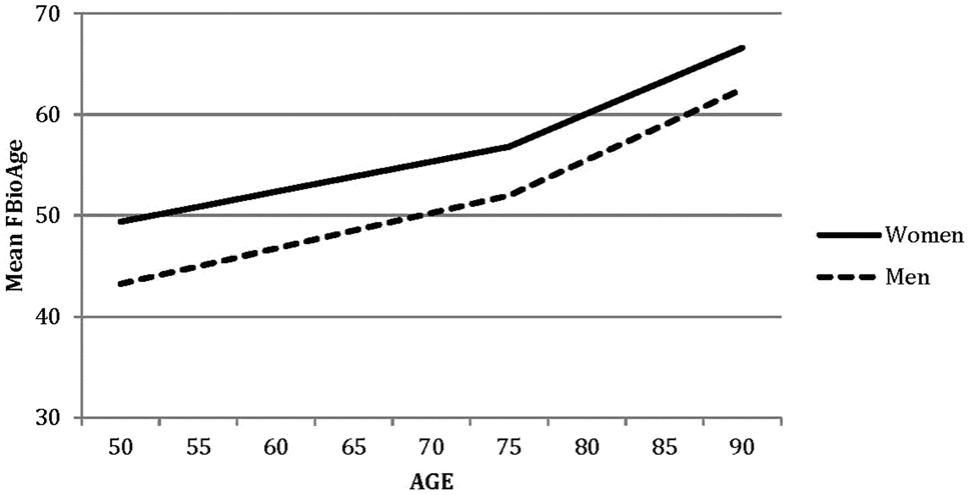
Changes in mean fBioAge estimated by the growth curve model

In the second phase of model fitting, additional models were tested to identify the nature of the sex differences in change trajectories; results are presented in the middle of Table 2. Using model 4 as the new baseline model, sex differences in each growth curve parameter were tested separately in models 5, 6, and 7. Comparing model fit statistics to model 4 indicated significant sex differences in intercept, only. Thus, men and women differ in mean fBioAge, but there are no significant sex differences in rates of change either before or after ChronAge 75. Change trajectories estimated by the growth curve model are presented in Fig. 2; growth curve parameter estimates are reported in Table 3. For both men and women, rates of change after ChronAge 75 are twice as fast as rates of change before ChronAge 75. For example, slope 1 for fBioAge is 0.32 for men while slope 2 is 0.78. The fact that two-slope models provide a significantly better fit to the data than one-slope models (*p*’s < 0.001) verifies that the difference in rates of change before and after ChronAge 75 was significant. The mean fBioAge at ChronAge 75 is five points higher for women than for men. Because slopes estimated for men were slightly greater than the slopes estimated for women, the change trajectories for men and women converge slightly, but not significantly, over the age range: the sex difference at ChronAge 50 is 6.11 whereas it is 4.10 at ChronAge 90.

**Table 3:** Parameters estimates (confidence intervals) from biometric growth curve model 9

Parameter	Estimate	C.I.
Intercept – Men	51.70	(50.82, 52.59)
Slope 1 – Men	0.32	(0.27, 0.38)
Slope 2 – Men	0.78	(0.58, 0.96)
Intercept – Women	56.81	(56.16, 57.58)
Slope 1 – Women	0.29	(0.25, 0.34)
Slope 2 – Women	0.64	(0.49, 0.77)
Ai → Intercept	2.47	(1.43, 3.46)
Ei → Intercept	3.05	(2.24, 3.88)
Ai → Slope 1	0.01	(−0.05, 0.10)
Ei → Slope 1	0.05	(−0.01, 0.12)
As1 → Slope 1	0.00	(0.00, 0.11)
Es1 → Slope 1	0.00	(0.00, 0.12)
Ai → Slope 2	0.23	(−0.16, 0.42)
Ei → Slope 2	−0.21	(−0.36, 0.09)
As1 → Slope 2	0.01	(−0.50, 0.48)
Es1 → Slope 2	0.35	(−0.59, 0.54)
As2 → Slope 2	0.01	(−0.48, 0.43)
Es2 → Slope 2	−0.17	(−0.54, 0.56)

### Twin analysis

The purpose of the twin analysis was to determine whether A, C, and S influences on the growth curve parameters were significant. Therefore, three additional models were fit to the data; results are reported in the bottom section of Table 2. In model 8, additive genetic effects (A) were dropped from the model, which did not have a significant impact on model fit versus model 4. In model 9, both correlated environmental (C) and shared environmental (S) effects were dropped from the model, with no significant change in model fit versus model 4. In model 10, A, C, and S were all dropped from the model, which resulted in a significant change in model fit versus model 4. Comparison of model 9 with model 10 provides the most direct test of genetic influences on the growth curves, and the difference in model fit was significant (change in fit = 26.7, df = 6, *p* < .001). Thus, model fitting indicated significant genetic influences on fBioAge.

**Fig. 3:**
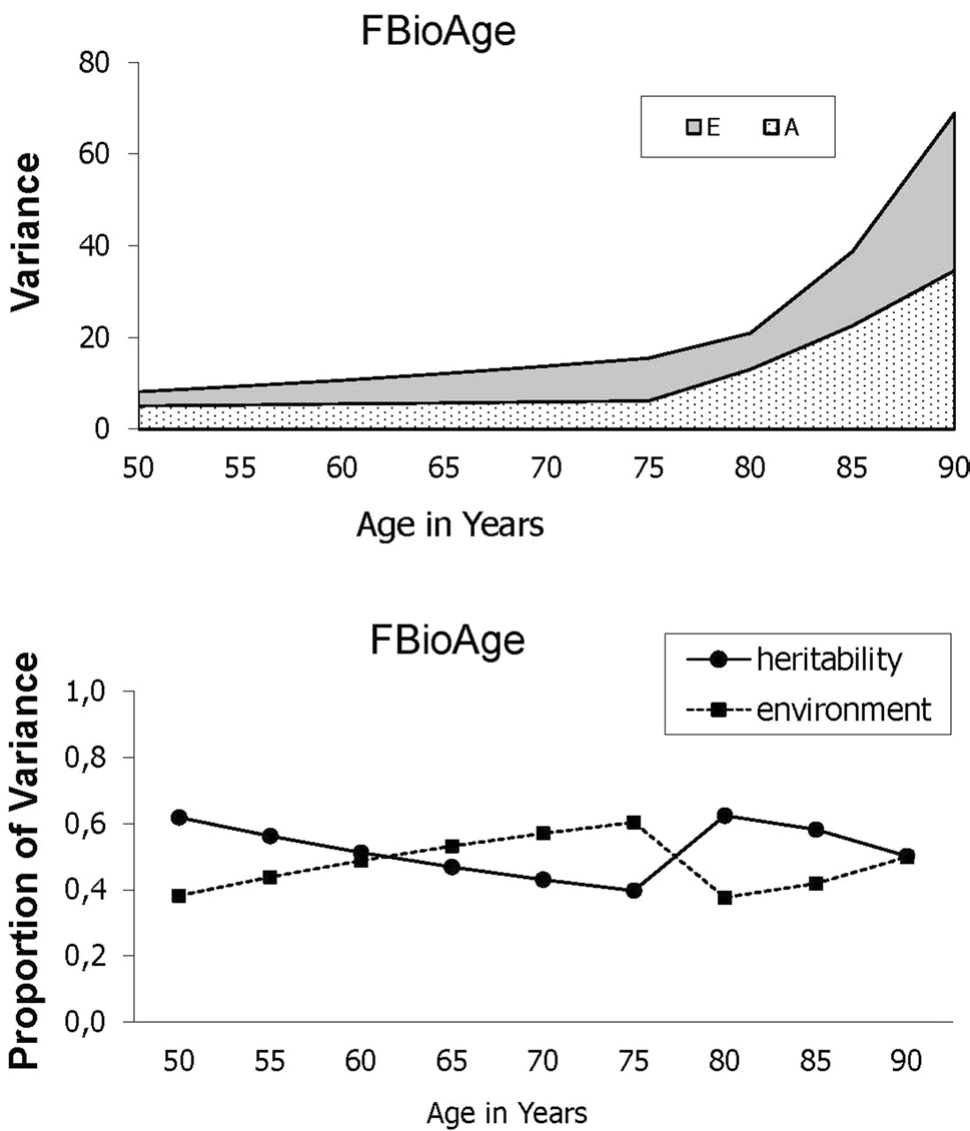
Changes in genetic and environmental components of variance for fBioAge estimated by the growth curve model

Changes in genetic and environmental components of variance for fBioAge estimated by model 9 are presented in Fig. 3: both raw variances (top) and proportions of variance (bottom). Total variance in fBioAge was fairly flat (about 20) up until ChronAge 75, at which point total variance started to increase dramatically, reaching a maximum of 69 at ChronAge 90. The bottom half of Fig. 3 indicates that the increase in total variance was fairly evenly split between genetic and environmental variance. In addition to estimating changes in genetic and environmental influences on fBioAge, the biometric latent growth curve model provides a means for identifying the nature of the genetic and environmental influences. Biometric parameter estimates resulting from model 9 are reported in Table 3. Parameter estimates for the paths from Ai to intercept and Ei to intercept were significant; however, genetic influences on slopes were not significant. Thus, although there were significant genetic influences on mean level of fBioAge, rates of change were influenced primarily by nonshared environmental factors.

## Discussion

ChronAge, an indicator of age used in most aging studies, is primarily an index of time. fBioAge is a promising alternative to ChronAge, being an age measure that also can be considered as an indicator of aging, per se. fBioAge consists of functional body measures, i.e. muscle strength, lung functioning, gait, and sensory abilities (Anstey et al. 1996). The biological age variable developed within our group was based on the concept of fBioAge (Anstey 2008), and has recently been used in a cross-sectional setting (Wahlin et al. under review). The current study is unique in its longitudinal approach to examination of fBioAge. We found that the average trajectories of fBioAge across time increase with a rather low steady rate up to 75 chronological years of age, and after that the rate of change is twice as fast. This type of increase in the trajectories was the same for both women and men. However, the intercept of the LGCM for women was significantly higher than the corresponding intercept for men. The individual differences (i.e. total variance) increased strongly after ChronAge 75. The SATSA twin study design allowed us to examine genetic and environmental influences on the trajectories. We found that genetic and environmental factors have an approximately equal influence on the variance.

It is in line with our first hypothesis that the trajectories of fBioAge increase with different rates across the life span. fBioAge has, in this respect, an important characteristic as an aging indicator that ChronAge does not have. The aging processes goes on at individual rates, and Nakamura and Miyao (2008) also observed that the rate of change in biological age increases later in life, although in their study it was from 65 chronological years of age. Our results are also in line with other longitudinal research on different types of body performance measures (such as cognition and grip strength) that do not change that much before ChronAge 65–70, after which the change increases (e.g., Sternäng et al. 2015a, b). Also, the variance of fBioAge is rather high in old ChronAge, which means that the individual trajectories differ a lot. Individual differences in aging can be captured by fBioAge but not by ChronAge, which has the same rate of change for all individuals. This is also an important advantage for fBioAge compared to ChronAge as aging indicator.

We found gender differences in the fBioAge trajectories. This was only partly in line with the second hypothesis, since the differences were only observed in levels (intercepts) and not in change across time. Women had higher fBioAge than men across the studied ChronAge range in this sample of Swedish twins. This type of gender difference was also found in cross-sectional data from Bangladesh (Wahlin et al. under review). Other results from other countries have also shown higher fBioAge for women (e.g., Anstey et al. 1996; Nakamura and Miyao 2008). In Bangladesh, this was expected, since the life situation for older women connected to health and possibilities are worse than for men (e.g., Herlitz and Kabir 2006). In Sweden, however, it is not as easy to understand why women have higher fBioAge than men, since women, for example, live longer than men in general in Sweden and in nearly all other countries (United Nations Statistics Division 2013). However, while women live longer they also report having worse health than men do (Brayne et al. 2001) which probably influences strongly their fBioAge level. This paradox and the gender differences in longevity and aging are not yet fully understood (Austad 2006; Regan and Partridge 2013), and these different levels of fBioAge in women and men need further study. However, we found no significant gender differences in the rate of change in fBioAge, which is different to findings showing that women may have a slower rate of aging than men (Nakamura and Miyao 2008). To be sure, the curves for men and women in the present study seem to converge somewhat in late adulthood, but that tendency was not statistically significant.

We observed that the levels of fBioAge, for this population, were rather equally influenced by genetic and environmental factors. There was a nonsignificant trend for increasing environmental variance after ChronAge 75. This result is in line with our hypothesis that environmental factors increase their influence during the life span. We also observed that it was the nonshared environmental factors primarily that influenced the rate of change in fBioAge. Other studies have also shown that several traits demonstrate increased nonshared environmental variance in old age (Finkel et al. 2015; Finkel and Reynolds 2010; Gavrilova and Gavrilov 2009). Still others have found that heritability remains stable, at least up to 80 years of age (McGue and Christensen 2013). Since environmental factors include things that a person can affect, such as lifestyle, type of work, and living place, it might be possible to influence our own fBioAge development to a certain extent, for example, with change of lifestyle. An increase in nonshared environmental variance could result from the cumulative effect of (distal) lifestyle factors experienced across adulthood. If so, then interventions should focus on lifestyle changes in early and midlife. On the other hand, an increase in nonshared environmental variance could result from proximal environmental events unique to the individual (illness or injury) from which the individual, as a result of reduced resilience, is no longer able to recover in a timely fashion. In that case, interventions might better be focused on the present situation. Additional research will be required to locate the optimal focus of interventions. The potential to reduce fBioAge in late life is an interesting challenge that needs further examination. Since there were no gender differences in the influence of genetic and environmental factors, this could potentially be equally possible for men and women.

## Limitations

Even if this study has strong features, it also has some limitations worth noticing. First, since the fBioAge levels were different for men and women, we cannot exclude that this difference partly has a methodological reason. However, we controlled the fBioAge variable for sex in the underlying measures, i.e. grip strength was adjusted for sex and lung capacity for body volume. Limitations include many of the statistical assumptions common to structural equation models. The data are assumed to be missing at random and the sample is assumed to be relatively homogeneous. As one focus of the current analysis was on sex differences, it is important to note that patterns of participation and attrition did not differ significantly for men and women. As with any longitudinal sample, attrition occurred in the SATSA sample. However, using an age-based growth curve model instead of a time-based model allowed us to maximize power, especially for twin pairs with more participation waves. Finally, although fBioAge demonstrated positive skew, the use of the two-slope growth curve model allowed us to capture the increase in individual differences after ChronAge 75.

## Conclusions

This study is unique by its examination of longitudinal trajectories in fBioAge. The rate of increase in fBioAge was twice as fast after ChronAge 75. There were gender differences in the trajectories. fBioAge was significantly higher in women than in men, but there were no gender differences in rates of change with ChronAge. fBioAge is fairly equally influenced by genetic and environmental factors. In addition, the rates of change in fBioAge were primarily due to non-shared environmental factors. The different rates of aging, and the individual differences in aging that we observed in this study, can be captured by fBioAge but not by ChronAge, which assumes the same rate of aging for all individuals. These features are important advantages for fBioAge compared to ChronAge as an aging indicator. In the future, we will examine further how fBioAge, and in particular the distribution of fBioAge in adults over ChronAge 75, relates longitudinally to aging-sensitive functions such as cognitive abilities.
